# Association of Regular Endoscopic Screening with Interval Gastric Cancer Incidence in the National Cancer Screening Program

**DOI:** 10.3390/jcm11010230

**Published:** 2021-12-31

**Authors:** Choong-Kyun Noh, Eunyoung Lee, Gil Ho Lee, Sun Gyo Lim, Bumhee Park, Sung Jae Shin, Jae Youn Cheong, Kee Myung Lee

**Affiliations:** 1 Department of Gastroenterology, Ajou University School of Medicine, Suwon 16499, Korea; cknoh23@gmail.com (C.-K.N.); micorie@hanmail.net (G.H.L.); mdlsk75@gmail.com (S.G.L.); shsj9128@ajou.ac.kr (S.J.S.); jaeyoun620@gmail.com (J.Y.C.); 2 Department of Biomedical Informatics, Ajou University School of Medicine, Suwon 16499, Korea; eylee@aumc.ac.kr (E.L.); bhpark@ajou.ac.kr (B.P.); 3 Office of Biostatistics, Ajou Research Institute for Innovative Medicine, Ajou University Medical Center, Suwon 16499, Korea; 4 Department of Medical Sciences, Biomedical Informatics, Graduate School of Ajou University, Suwon 16499, Korea

**Keywords:** gastric cancer, interval cancer, screening, endoscopy, national cancer screening program

## Abstract

Although regular endoscopic screening may help in early detection of gastric cancer, interval cancer remains a problem in the screening program. This study evaluated the association between regular endoscopic screening and interval cancer detection in the Korean National Cancer Screening Program (KNCSP). We defined three groups (regularly, irregularly, and not screened) according to the screening interval, and the trends in the interval cancer rate (ICR) between the groups were tested using the Cochran–Armitage test. The influence of regular endoscopic screening on the risk of interval cancer was evaluated using multivariable logistic regression. Among the 11,642,410 participants who underwent endoscopy, the overall ICR was 0.36 per 1000 negative screenings. The ICR of the not screened group (0.41) was the highest among the three groups and the risk of interval cancer in this group was 1.68 times higher (*p* < 0.001) than that in the regularly screened group. Women in their 40s who had regular screening with no history of intestinal metaplasia and gastric polyps would have the lowest probability of having interval cancer (0.005%). Regular participation in endoscopic screening programs for reducing the risk of interval cancer may help to improve the quality of screening programs.

## 1. Introduction

Gastric cancer is considered as an important contributor to the global cancer burden, accounting for the fifth highest incidence among cancers and the third highest cause of cancer-related mortality [1]. In 2018, there were over 1,000,000 new patients diagnosed with gastric cancer and 783,000 deaths [1]. Particularly, the incidence of gastric cancer is high in Eastern Asia, including South Korea and Japan [1].

Generally, early gastric cancer involves small-sized lesions with no apparent symptoms. Once the disease progresses because of the late detection of the lesion, the mortality of cancer patients increases. Hence, it is crucial to detect gastric cancer in the early stage, followed by appropriate treatments, for which some Asian countries have operated screening programs for gastric cancer. Some studies have reported that a screening program reduced gastric cancer-related mortality [2,3,4]. However, the efficiency of the screening program is still controversial [5,6].

Screening programs need to detect cancer early and control screening quality. A significant factor to determine the quality of screening programs is to achieve a reduction in interval cancer [7]. Interval cancer refers to cancer that was negative in the screening test and was detected before the next screening surveillance examination [7]. While upper endoscopy is highly useful for the early detection of gastric cancer [8,9,10], interval cancer is an issue that needs to be solved in the screening program.

Unlike colonoscopy, which has predictors for interval cancer risk [7], there is no effective way for an upper endoscopy to reduce the incidence of interval cancer because it lacks clear quality indicators. Screening programs are composed of a survey on baseline information and examinations for detecting cancer. The survey questionnaire includes the examination history. We hypothesized that regular participation in a screening program, rather than irregular participation would reduce the incidence of interval cancer by providing endoscopists with accurate information, thus increasing the accuracy of the examination. Accordingly, this study aimed to evaluate risk factors of interval cancer in screening participants and to assess reductions in the number of interval gastric cancer cases with a two-year interval of regular endoscopic screening using the large cohort database from the Korean National Cancer Screening Program (KNCSP). Additionally, we report the incidence probability of interval cancer by the identified risk factors.

## 2. Methods

### 2.1. Study Population

This is a retrospective, large population-based cohort study using the KNCSP database. The total cohort was selected from the two most recent KNCSP cycles that we obtained: one cycle of 2013–2014 and the other cycle of 2015–2016. The data of the first cycle was used as the reference cohort for confirming the consecutive screening of participants. Based on this data and the answers to the questionnaire of history of endoscopy from the data of the second cycle, the cohort of the second cycle was divided into three groups: the regularly screened group (group 1) was defined as those who reported their last endoscopy within the past two years; the irregularly screened group (group 2) was defined as those who reported their last endoscopy within the past 2–10 years; and the not screened group (group 3) was defined as those who reported no endoscopy in the past ten years or no experience in their lifetime. Individuals who newly participated in the 2015–2016 cycle were also divided into three groups according to their endoscopy history with/without KNCSP invitation. Exclusion criteria for participants were as follows: (1) previously diagnosed gastric cancer; (2) upper gastrointestinal (UGI) series only; (3) disqualified from the KNCSP; (4) did not return for subsequent screening; (5) participants who received a gastric cancer diagnosis in the 2013–2014 cycle; and (6) participants who received negative results but were diagnosed with ulcer were confirmed subsequent gastric cancer diagnosis within eight weeks. The study protocol was approved by the Ajou University hospital’s institutional review board (approval No. AJIRB-MED-MDB-19-109), which waived the requirement for individual informed consent owing to the use of a de-identified dataset.

### 2.2. The KNCSP and Data Collection

The specific KNCSP protocol is described in the Supplement method [11,12]. Participant data were extracted from the KNCSP database in 2015–2016. The KNCSP data included demographic characteristics, a brief history of endoscopy, and medical history using questionnaires; endoscopy, biopsy, and comprehensive cancer screening results; and screening sites and providers. We defined screening results as positive if endoscopic results were recorded as possible gastric cancer, early gastric cancer, or advanced gastric cancer, or if biopsy results were recorded as low-grade dysplasia, high-grade dysplasia, suspicious gastric cancer, or gastric cancer. We tracked and checked interval gastric cancer cases up to 31 December 2017 by linking individual medical records from the National Health Insurance Sharing Service-National Health Information Database (NHIS-NHID). We defined interval gastric cancer when participants received a diagnosis code for gastric cancer (International Classification of Diseases, 10th revision, C16.xx) within one year of endoscopy screening (negative screening results) [13,14]. Missed cancer is an important component of interval cancer that determines screening quality. Therefore, the observation period to detect interval cancer was defined as “within one year” of negative screening results. Interval cancer was found by additional examinations performed at different centers from the screening program for various reasons, including symptom development.

### 2.3. Statistical Analyses

Participants’ demographics, medical history, and screening characteristics between the three groups were summarized and compared using the chi-squared test. The interval cancer rate (ICR) per 1000 negative screenings was computed as the number of interval cancer divided by the number of negative screenings and was presented with 95% confidence intervals (CIs). The trend in the ICR was tested with a one-sided Cochran–Armitage test. The multivariable logistic regression analysis was conducted for identifying risk factors associated with interval cancer. Additionally, a stepwise selection method was used for selecting the best subset of risk factors for predicting interval cancers among the risk factor candidates, including screening regularity (three groups), sex, age groups, and history of gastric diseases. The odds ratios (ORs) were provided with the corresponding 95% CIs. Based on the estimated parameter of multivariable logistic regression analysis, the probability of interval cancer was also predicted. All the reported *p*-values were two-sided, and *p*-values of <0.05 were considered statistically significant. Analyses were performed using SAS version 9.4 (SAS Institute Inc., Cary, NC, USA).

## 3. Results

### 3.1. Study Population and Baseline Characteristics

**Figure 1:**
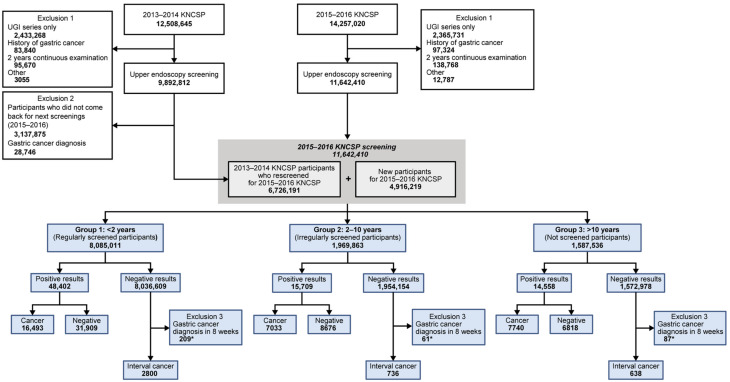
Flow diagram for selection of enrolled participants in this study. Among the participants from the first cycle (9,892,812, 2013–2014), 6,726,191 (67.99%) were re-screened in the next cycle of 2015–2016, and 3,137,875 (31.72%) did not return for screening. In the second cycle, 4,916,219 were newcomers who did not participate in the previous cycle. KNCSP, Korean National Cancer Screening Program. * Participants who received negative results but were diagnosed with ulcer were confirmed subsequent gastric cancer diagnosis within eight weeks.

The total cohort comprised of 21,535,222 participants who underwent endoscopy in the screening program between 2013 and 2016 (mean [standard deviation] age, 55.61 [10.61] years; 11,761,709 [54.62%] women). Among them, 9,892,812 individuals (45.94%) participated in the first cycle of 2013–2014 and 11,642,410 (54.06%) in the second cycle of 2015–2016 (Figure 1). Participants were divided into three groups based on screening intervals and regularity. The regular screened group (n = 8,085,011, 69.44%) was the most prevalent, followed by the irregular screened group (n = 1,969,863, 16.92%) and the not screened group (n = 1,587,536, 13.64%). All three groups had differences in all the characteristics (*p* < 0.001 for all). There were more men and younger (40–49 years) participants in the not screened group than in other screened groups. Most notably, history of gastric diseases, including atrophic gastritis, intestinal metaplasia, ulcer, and gastric polyp, were more common in the regularly screened group than in other groups (Table 1). The overall gastric cancer detection rates of the 2013–2014 cycle and 2015–2016 cycle were 0.29 (per 100, 95% CI, 0.17–0.29) and 0.27 (per 100, 95% CI, 0.26–0.27), respectively (Supplementary Table S1).

### 3.2. ICR and Their Risk Factors in the National Cancer Screening Program

In the cycle of 2015–2016, 4174 participants were diagnosed with gastric cancer within one year of negative screening. Overall, the ICR in this cycle was 0.36 per 1000 negative screenings (95% CI, 0.35–0.37). The ICR for the not screened group (0.41, 95% CI, 0.37–0.44) was the highest among the three groups, which was 1.17 times higher than those for the regularly screened group (0.35, 95% CI, 0.34–0.36). Based on the results of the Cochran–Armitage test for trend, there was an increasing trend in ICR between the three groups (*p* < 0.001) (Table 2).

The multivariable logistic regression analysis was conducted to identify risk factors associated with interval cancer. The risk factors of screening regularly, sex, age groups, and the presence of intestinal metaplasia and gastric polyps were selected from the stepwise selection method, and they were all significant (*p* < 0.001 for all). The risk of having interval cancer in the not screened group was 1.68 times higher (95% CI, 1.54–1.84) than that in the regularly screened group. As the participants aged, the OR of interval cancer gradually increased from 2.63 (50–59 years; 95% CI, 2.31–2.99) to 14.09 (≥80 years; 95% CI, 11.93–16.65). Men (OR: 2.58; 95% CI, 2.40–2.77), the history of intestinal metaplasia (OR: 1.99; 95% CI, 1.48–2.69), and gastric polyp (OR: 2.44; 95% CI, 2.09–2.86) were significantly associated with interval cancer (Table 3).

### 3.3. The Probability Model for Having Interval Gastric Cancer after Endoscopic Screening

We developed a probability model of interval gastric cancer after endoscopic screening based on the analyzed data. Based on the multivariable logistic regression results, the probability of having interval cancer was computed as:

$$\mathrm{Pr}\left(interval\;cancer\right)=\frac{\exp\left(A\right)}{1+\exp\left(A\right)}$$

where

$$A=-9.991+0.236x_{group2}+0.520x_{group3}+0.947x_{male}\;\;+0.966x_{age50s}+1.693x_{age60s}+2.295x_{age70s}\;\;+2.646x_{age80s}+0.689x_{intestinal\;metaplasia}\;+0.894x_{polyp}$$

$x_{group2}=1$ for the irregularly screened group, otherwise 0

$x_{group3}=1$ for the not screened group, otherwise 0

$x_{male}=1$ for male, otherwise 0

$x_{age50s}=1$ for 50–59 years age group, otherwise 0

$x_{age60s}=1$ for 60–69 years age group, otherwise 0

$x_{age70s}=1$ for 70–79 years age group, otherwise 0

$x_{age80s}=1$ for ≥80 years age group, otherwise 0

$x_{intestinal\;metaplasia}=1$ for the presence of intestinal metaplasia, otherwise 0

$x_{polyp}=1$ for the presence of gastric polyps, otherwise 0.

Hence, participants who did not undergo endoscopy for cancer screening for >10 years were men in their 80s whom had both intestinal metaplasia and gastric polyps; these participants would have the highest probability of having interval cancer (1.350%). Meanwhile, those who had regular cancer screenings were women in their 40s who did not have intestinal metaplasia and gastric polyps; these participants would have the lowest probability (0.005%). Parameter estimates and its 95% CIs from the multivariable logistic regression are presented in Table 4.

## 4. Discussion

The interval of an endoscopic screening program directly influences gastric cancer-related survival [15], and individuals with a regular screening interval of <2 years tended to get diagnosed early, thereby being eligible for endoscopic treatment. In contrast, those who had a longer screening interval were more likely to be diagnosed with advanced stages of cancer where endoscopy was not effective [16]. Despite such reports on the relationship between the detection of gastric cancer and screening interval, there has been no study on the relationship between screening programs and interval cancer, which is a quality index of the screening program. With a large national cohort, we found that regular participation in the national cancer screening program was likely to reduce interval cancer. Even with endoscopic screening, the incidence of interval cancer increased when it was performed in a long screening interval (screening interval of <2 years vs. 2–10 years vs. >10 years). Particularly, the OR of those who had no endoscopic screening for 10 years was 1.68 times higher than that of participants who had screening every two years. Additionally, the risk factors for interval cancer were male gender, older age, and the presence of intestinal metaplasia and gastric polyps, based on which a probability model of interval cancer was developed. While the role of endoscopists during endoscopic screening is important, these results obtained using the large cohort database suggested that regular participation in screening should also be considered important.

Gastric cancer is histopathologically evaluated using biopsy of lesions detected with endoscopy, resulting in a definitive diagnosis [17,18]. Endoscopy has high diagnostic accuracy for the detection of gastric cancer [19]. Since it is difficult to suspect gastric cancer owing to the uncertainty of its symptoms in the early stage, endoscopy can play a significant role in the early detection of cancer. Thus, the endoscopic screening program has been operated for gastric cancer. When cancer is detected in an advanced stage, the prognosis of the patient would be poor; hence, the screening program aims to detect and treat cancer in the early stage. Case–control studies have reported that endoscopic screening could reduce gastric cancer-related mortality [3,4,20]. It is expected that endoscopy would be considered more important for the early detection of gastric cancer. In an endoscopic examination that allows direct observation of lesions and biopsy, the occurrence of interval cancer is a significant issue. Interval cancer includes missed lesions and latent lesions, whose number should be reduced by the screening program [21]. Since interval gastric cancer is a new terminology used in countries like South Korea and Japan, where nationwide screening programs for gastric cancer were established, there have been few studies on this subject. Previously, a single-center study characterized interval gastric cancer, found tumor location (lower body), and observed tumor differentiation [22]. However, this study comparatively analyzed interval cancer and the control with a small sample size in a single center; hence, it had a limitation in identifying risk factors.

In our study, the OR of interval cancer increased in participants who irregularly participated in the screening program, who were men, with older age, and with the presence of metaplasia and polyp history. With endoscopy, intestinal metaplasia was found to have irregular surfaces, such as ash-colored nodular change, plaque, patch, a rough mucosal surface, and villous appearance [23,24]. As for missed cancer, with upper endoscopy, unlike colonoscopy, it may be difficult to visually identify early gastric cancer and adenoma owing to irregular surfaces of the gastric mucosa rather than blind spots, which might have led to missing those lesions. Intestinal metaplasia increases with age [25] and is one of the important risk factors for gastric cancer [26]. From such perspectives, our study also showed similar results, wherein male sex, older age, and intestinal metaplasia were associated with interval cancer in participants in the screening program. The presence of gastric polyp history was also an associated factor for interval cancer (OR 2.44). In our study, tumor characteristics of interval cancer could not be identified owing to privacy issues of the KNCSP. It was also impossible to investigate whether polyps progressed to cancer because polyp history, like other factors, was obtained from the questionnaire provided to participants of the KNCSP. A previous study showed that gastric polyps could be a risk factor for cancer [27]; however, our results should be interpreted carefully. Additionally, the relationship between the number of polyps and cancer could not be evaluated. Thus, this relationship should be further investigated if polyp history itself is associated with the occurrence of interval cancer.

Although regular screening can be helpful for the early detection of gastric cancer, it remains unclear why it reduces interval cancer. The KNCSP was composed of a survey and an examination. In a pre-examination survey, previous endoscopic results and history need to be answered in detail, which can be checked by an endoscopist before the examination. Thus, it is speculated that such information of individuals who regularly participated would be more accurate than those who had no regular screening. Such pre-test information would be helpful for the endoscopic exam. Additionally, it is postulated that regular participation in screening would increase the chance of having an examination in the same institution. In such cases, endoscopists could check the previous endoscopic results and they might be able to perform the tests with more accurate information. Moreover, it is possible that individuals who regularly participate in screening are highly interested in their health and might be better in managing their health. However, such reasons cannot explain the association between interval cancer and regular screening. A prospective randomized study would show accurate results; however, it is practically difficult and may be accompanied by ethical issues, which makes it difficult to apply to this study.

There were some limitations to our study. First, the cancer information of individual patients was protected from being disclosed because of privacy issues; hence, it could not be used for analysis. Second, we could not analyze the survival rates associated with cancer stages and their effects in this study due to data unavailability. Third, *Helicobacter pylori* infection plays a significant role in gastric cancer onset [28]. Nevertheless, we were unable to identify the baseline status of *Helicobacter pylori* infection. Fourth, a history of UGI series was not considered. Fifth, our study utilized the KNCSP data of 2013–2016 and investigated up to 2017 to detect the occurrence of interval cancer. Despite limitation in representativeness, it was the most recent accessible data containing a large cohort of 11,642,410 participants who underwent endoscopic screening. Sixth, since several endoscopists participated in the KNCSP, differences in endoscopy quality may exist. Thus, Korea is overcoming this limitation by implementing a quality control program led by the society and government. Seventh, we did not confirm the exact preparation status during screening endoscopy. Finally, potential risk of recall bias exists, as questionnaires were used for data collection.

In summary, using a nationwide cohort, we investigated the baseline characteristics that increased the risk of interval cancer in participants of the screening program. Although guidelines exist to improve the quality of screening programs by increasing the number of endoscopies performed [29,30], interval cancer remains an important issue. To reduce interval cancer, it is important not only to have quality control by endoscopists but also to ensure that regular screening of participants is performed. Based on our results, it is expected that active participation is required to improve the quality of the screening program, and endoscopists should refer to participants’ baseline information in clinical practice to reduce the incidence of interval cancer.

## Figures and Tables

**Table 1:** Sociodemographics of the screening participants and characteristics of screenings based on participation interval for screening endoscopy.

Characteristics	Group 1 (<2 Years) *n* = 8,085,011	Group 2 (2–10 Years) *n* = 1,969,863	Group 3 (>10 Years) *n* = 1,587,536	*p* Value ^a^
Sex, No. (%)				<0.001
Male	3,629,799 (44.90)	888,042 (45.08)	816,937 (51.46)	
Female	4,455,212 (55.10)	1,081,821 (54.92)	770,599 (48.54)	
Age (year), No. (%)				<0.001
40–49	2,190,059 (27.09)	696,934 (35.38)	800,614 (50.43)	
50–59	2,635,522 (32.60)	640,236 (32.50)	442,263 (27.86)	
60–69	2,113,188 (26.14)	389,015 (19.75)	220,777 (13.91)	
70–79	1,002,090 (12.39)	198,231 (10.06)	98,010 (6.17)	
≥80	144,152 (1.78)	45,447 (2.31)	25,872 (1.63)	
Hospital type, No. (%)				<0.001
General hospital (≥100 beds)	2,444,925 (30.24)	529,594 (26.88)	495,716 (31.23)	
Hospital (30–99 beds)	1,459,087 (18.05)	392,878 (19.94)	333,562 (21.01)	
Clinics (<30 beds)	4,180,999 (51.71)	1,047,391 (53.17)	758,258 (47.76)	
Screening location, No. (%)				<0.001
Capital area ^b^	3,955,485 (48.92)	936,039 (47.52)	828,406 (52.18)	
Non-capital area	4,129,526 (51.08)	1,033,824 (52.48)	759,130 (47.82)	
History of gastric disease ^c^, No. (%)				
Atrophic gastritis	1,169,183 (14.46)	194,925 (9.90)	53,616 (3.38)	<0.001
Intestinal metaplasia ^d^	77,059 (0.95)	5392 (0.27)	1686 (0.11)	<0.001
Ulcer	795,076 (9.83)	150,153 (7.62)	62,044 (3.91)	<0.001
Gastric polyp	227,829 (2.82)	33,069 (1.68)	7166 (0.45)	<0.001
Other	824,317 (10.20)	182,383 (9.26)	39,521 (2.49)	<0.001

^a^*p* Values were calculated by chi-squared test; ^b^ The capital area includes Seoul, Incheon, and Gyeonggi province; ^c^ The source of these variables was participants’ self-reported questionnaires for the National Cancer Screening Program; ^d^ Intestinal metaplasia was accompanied by atrophic gastritis.

**Table 2:** Overall interval cancer rates with 95% confidence intervals arranged group-wise.

Variable	Number	Negative Screening	Interval Cancer	ICR Per 1000 Negative Screenings (95% CI)	*p* Value ^a^
Overall	11,642,410	11,563,741	4174	0.36 (0.35 to 0.37)	N/A
Group 1 (regular rescreened group, <2 years)	8,085,011	8,036,609	2800	0.35 (0.34 to 0.36)	<0.001
Group 2 (irregular screened group, 2–10 years)	1,969,863	1,954,154	736	0.38 (0.35 to 0.40)	
Group 3 (not screened group, >10 years)	1,587,536	1,572,978	638	0.41 (0.37 to 0.44)	

Abbreviations: ICR, interval cancer rates, CI, confidence intervals; ^a^ *p* Values were calculated using the Cochran–Armitage test for trend.

**Table 3:** Multivariable logistic regression analysis of risk factors associated with interval cancer detection in the Korean National Cancer Screening Program for gastric cancer.

Variable	OR (95% CI)	*p* Value ^a^
Group		<0.001
Group 1 (regular screened group, <2 years)	1	
Group 2 (irregular screened group, 2–10 years)	1.27 (1.16 to 1.38)	
Group 3 (not-screened group, >10 years)	1.68 (1.54 to 1.84)	
Sex		<0.001
Female	1	
Male	2.58 (2.40 to 2.77)	
Age group, years		<0.001
40–40	1	
50–59	2.63 (2.31 to 2.99)	
60–69	5.44 (4.81 to 6.15)	
70–79	9.93 (8.75 to 11.26)	
≥80	14.09 (11.93 to 16.65)	
History of intestinal metaplasia ^b,c^		<0.001
Absent	1	
Presence	1.99 (1.48 to 2.69)	
History of Gastric polyp ^b^		<0.001
Absent	1	
Presence	2.44 (2.09 to 2.86)	

Abbreviations: CI, confidence interval; OR, odds ratio; ^a^ *p* Values were calculated using the Wald chi-square test. The multivariable logistic model was selected using the stepwise selection method with group, sex, age group, gastric ulcer, atrophic gastritis, intestinal metaplasia, and gastric polyps. ^b^ The source of these variableswas participants’ self-reported questionnaires for the National Cancer Screening Program; ^c^ Intestinal metaplasia was accompanied by atrophic gastritis.

**Table 4:** Parameter estimates and 95% confidence intervals from multivariable logistic regression analysis estimating the probability of having interval gastric cancer after screening endoscopy.

Parameter	Estimate (95% CI)	*p* Value
Intercept	−9.99 (−10.12 to −9.87)	<0.001
Group 2 (irregularly screened group, 2–10 years)	0.24 (0.15 to 0.32)	<0.001
Group 3 (not−screened group, >10 years)	0.52 (0.43 to 0.61)	<0.001
Male	0.95 (0.88 to 1.02)	<0.001
Age group (50–59)	0.97 (0.84 to 1.10)	<0.001
Age group (60–69)	1.69 (1.57 to 1.82)	<0.001
Age group (70–79)	2.30 (2.17 to 2.42)	<0.001
Age group (≥80)	2.65 (2.48 to 2.81)	<0.001
History of intestinal metaplasia ^a,b^	0.69 (0.39 to 0.99)	<0.001
History of gastric polyp ^a^	0.89 (0.74 to 1.05)	<0.001

Abbreviations: CI, confidence interval; OR, odds ratio. ^a^ The source of these variables was participants’ self-reported questionnaires for the National Cancer Screening Program; ^b^ Intestinal metaplasia was accompanied by atrophic gastritis.

## Data Availability

Data cannot be shared publicly because of the sensitive nature of the data collected for this study. Data are available from the Korea National Health Insurance Sharing Service (contact via https://nhiss.nhis.or.kr, contact: +82-33-736-2432 (ext. 2433)) for researchers who meet the criteria for access to confidential data.

## Supplementary Materials

The following supporting information can be downloaded at: https://www.mdpi.com/article/10.3390/jcm11010230/s1, Table S1: Overall Screening Performance for Gastric Cancer Between The 2013–2014 and 2015–2016 Korean National Cancer Screening Program Cyclesa.
