# Supplementary material for: Association of Regular Endoscopic Screening with Interval Gastric Cancer Incidence in the National Cancer Screening Program
Source: J Clin Med. 2021 Dec 31;11(1):230. doi: 10.3390/jcm11010230 (PMC8745904; doi:10.3390/jcm11010230)
Supplement: Supplementary file 1 [file jcm-11-00230-s001.zip › jcm-1512681-supplementary.pdf]

SUPPLEMENT

Supplemental Methods

1. The Korean National Cancer Screening Program (KNCSP)

The KNCSP provides screening services for six common cancers: the stomach, liver, colorectum, breast, uterine cervix, and lung. For gastric cancer, the KNCSP recommends biennial screening with either UGI series or upper endoscopy for men and women aged ≥40 years, who are invited for screening based on the birth year every two years (one cycle of the KNCSP). Eligible participants can voluntarily undergo screening at any nationally accredited screening center but are required to schedule screening only during their designated year (from January 1 to December 31). Upon completion of screening, the overall cancer screening results are mailed and are officially recorded according to five categories: (1) negative, (2) benign disease, (3) suspicious gastric cancer, (4) gastric cancer, and (5) others. When a histological diagnosis is made, gastric cancer is confirmed and recorded in the national cancer registry.

2. Targeted population with Korean National Cancer Screening Program for six common cancers

Stomach: aged 40 years or older,

Liver: aged 40 years or older with high-risk group of liver cancer,

Colorectum: aged 50 years or older,

Breast: aged 40 years or older,

Uterine cervix: aged 20 years or older,

Lung: aged 54-74 years with a high risk of lung cancer

3. Screening endoscopy protocol in the Korean National Cancer Screening Program

An endoscopic examination involves acquiring images after close observations by an endoscopist, for which imaging of at least eight areas (duodenum, ampulla, antrum, angle, corpus, fundus with retroflexion view, esophagogastric junction, and esophagus) is recommended, along with multiple additional images in areas with abnormal lesions. A tissue biopsy is performed when abnormal findings are detected in the endoscopic examination. If further evaluation or treatment is needed based on the biopsy or endoscopic findings, then the patient is referred to an upper-tier hospital for re-examination. If food materials were observed during endoscopy, which hinders accurate inspection of the gastric mucosa, endoscopy was terminated, and re-examination was conducted at the discretion of the endoscopists. Fluid was completely removed prior to observing the gastric mucosa for examination. The diagnosis of atrophy and metaplasia were based on the endoscopic findings. When necessary, the diagnosis was confirmed by histology. Atrophy was defined as visibility of the mucosal vascular pattern, while intestinal metaplasia was defined as replacement of the surface, foveolar, and glandular epithelium in the oxyntic or antral mucosa with intestinal epithelium.

The endoscopic results were reported according to nine categories: (1) negative, (2) gastritis, (3) possible gastric cancer, (4) early gastric cancer, (5) advanced gastric cancer, (6) gastric ulcer, (7) gastric polyp, (8) subepithelial tumor, and (9) others. As needed, a biopsy was performed, and results were reported according to eight categories: (1) negative, (2) gastritis, (3) inflammation or hyperplastic lesions, (4) low-grade dysplasia, (5) high-grade dysplasia, (6) suspicious gastric cancer, (7) gastric cancer, and (8) others. The cancer screening overall results were reported according to five categories: (1) negative, (2) benign disease, (3) suspicious gastric cancer, (4) gastric cancer, and (5) others.

Table S1. Overall Screening Performance for Gastric Cancer Between The 2013-2014 and 2015-2016 Korean National Cancer Screening Program Cycles[a]

**2013-2014 cycle**	
Sensitivity	0.798 (0.794-0.802)
Specificity	0.995 (0.995-0.995)
Positive predictive value	0.375 (0.371-0.378)
Detection rates (per 100)	0.291 (0.171-0.287)
Positive rates (per 1,000)	7.750 (7.695-7.804)
**2015-2016 cycle**	
Sensitivity	0.873 (0.870-0.877)
Specificity	0.996 (0.996-0.996)
Positive predictive value	0.397 (0.394-0.401)
Detection rates (per 100)	0.269 (0.266-0.272)
Positive rates (per 1,000)	6.757 (6.710-6.804)

[a] Presented with 95% confidence interval.
